# Cytonuclear discordance in the Florida Everglades invasive Burmese python (*Python bivittatu*s) population reveals possible hybridization with the Indian python (*P. molurus*)

**DOI:** 10.1002/ece3.4423

**Published:** 2018-08-19

**Authors:** Margaret E. Hunter, Nathan A. Johnson, Brian J. Smith, Michelle C. Davis, John S. S. Butterfield, Ray W. Snow, Kristen M. Hart

**Affiliations:** ^1^ U.S. Geological Survey Wetland and Aquatic Research Center Gainesville Florida; ^2^ Wetland and Aquatic Research Center Cherokee Nation Technologies Davie Florida; ^3^ U.S. National Park Service Everglades National Park Homestead Florida; ^4^ U.S. Geological Survey Wetland and Aquatic Research Center Davie Florida

**Keywords:** hybridization, invasive species, mitochondrial marker, nuclear microsatellite marker, phylogenetic population structure

## Abstract

The invasive Burmese python (*Python bivittatus*) has been reproducing in the Florida Everglades since the 1980s. These giant constrictor snakes have caused a precipitous decline in small mammal populations in southern Florida following escapes or releases from the commercial pet trade. To better understand the invasion pathway and genetic composition of the population, two mitochondrial (mtDNA) loci across 1,398 base pairs were sequenced on 426 snakes and 22 microsatellites were assessed on 389 snakes. Concatenated mtDNA sequences produced six haplotypes with an average nucleotide and haplotype diversity of *π* = 0.002 and *h *=* *0.097, respectively. Samples collected in Florida from morphologically identified *P. bivittatus* snakes were similar to published cytochrome oxidase 1 and cytochrome *b* sequences from both *P. bivittatus* and *Python molurus* and were highly divergent (genetic distances of 5.4% and 4.3%, respectively). The average number of microsatellite alleles and expected heterozygosity were *N*_A_ = 5.50 and *H*_E_ = 0.60, respectively. Nuclear Bayesian assignment tests supported two genetically distinct groups and an admixed group, not geographically differentiated. The effective population size (*N*_E_ = 315.1) was lower than expected for a population this large, but reflected the low genetic diversity overall. The patterns of genetic diversity between mtDNA and microsatellites were disparate, indicating nuclear introgression of separate mtDNA lineages corresponding to cytonuclear discordance. The introgression likely occurred prior to the invasion, but genetic information on the native range and commercial trade is needed for verification. Our finding that the Florida python population is comprised of distinct lineages suggests greater standing variation for adaptation and the potential for broader areas of suitable habitat in the invaded range.

## 1 INTRODUCTION

Understanding the processes driving invasion dynamics of non‐native species represents an important challenge for biologists and resource managers. Advancements in molecular tools and techniques have allowed for the delimitation of taxonomic units and genetic diversity, and identification of nonnative animals and plants in the absence of reliable morphological data (Bock et al., 2015; Darling, 2015; Serrao, Steinke, & Hanner, 2014). In many cases, only molecular information can elucidate the phylogeographic origin, transportation routes into nonnative ranges, and release history of nonnative species. Further, genetic tools can help identify source–sink population dynamics and movement pathways across invasion ranges for control and eradication efforts. Collectively, genetic characterization can inform management decisions and help to guide targeted removal efforts (Collins, Vazquez, & Sanders, 2002; Ficetola, Miaud, Pompanon, & Taberlet, 2008; Kolbe et al., 2007; McPhee & Turner, 2009; Stepien & Tumeo, 2006; Vidal, García‐Berthou, Tedesco, & García‐Marín, 2010).

Accurate and efficient identification and classification at the species level are necessary for invasive species management. For example, accurate species identification can indicate the required habitat types, diet (including prey species), intrinsic ecological constraints, and climatic suitability (Chown et al., 2015; Gotelli & Stanton‐Geddes, 2015; Pfeiffer, Johnson, Randklev, Howells, & Williams, 2016; Rissler & Apodaca, 2007). Population expansion capabilities or limitations can be assessed through knowledge of the species life history, population growth rates, and susceptibility to diseases. Further, once the invasive species has been correctly identified, putative range expansions can be predicted using ecological niche models based on both the native and invasive species ranges (Ikeda et al., 2017; Mainali et al., 2015).

Understanding the potential for hybridization of invasive species is critical because diversity can be increased through crossing of divergent groups prior to release or during sustained releases over time of genetically divergent individuals. Hybridization events can lead to increased diversity, fitness, and fecundity in the invasive population (Kolbe et al., 2004, 2007; Vidal et al., 2010). Further, hybrid vigor and environmental selection can result in improved adaptation to the novel environment and increased areas of climatic suitability (Hahn & Rieseberg, 2017; Roman & Darling, 2007). Deleterious mutations can also accumulate through outbreeding depression via negative dominance effects (Oakley, Ågren, & Schemske, 2015).

In this study, we investigated putative origins, potential for hybridization with congeners, and population structure within the invasive Burmese python (*Python bivittatus*) population in the Greater Everglades Ecosystem (GEE) in Florida, USA. This giant constrictor snake has been reproducing in southern Florida since approximately the mid‐1980s (Willson, Dorcas, & Snow, 2011). The cryptic nature of these snakes has limited detection and control efforts (Hunter et al., 2015; Reed et al., 2011), and the population has now expanded from Everglades National Park (ENP) into the eastern and western coasts of southern Florida and the Florida Keys (Dove, Snow, Rochford, & Mazzotti, 2011; Pittman et al., 2014; Snow, Brien, Cherkiss, Wilkins, & Mazzotti, 2007). Pythons are impacting the ecosystem through heavy predation on mesomammals, including imperiled species, resulting in extensive declines of formerly common species (Dorcas et al., 2012; McCleery et al., 2015; Reichert et al., 2017; Sovie, McCleery, Fletcher, & Hart, 2016).

*Python bivittatus* taxonomy and nomenclature have been uncertain in part due to the sympatric distribution with *P. molurus* in the native range and lack of a designated neotype (Jacobs, Auliya, & Böhme, 2009; Schleip & O'Shea, 2010). The species was first recognized by Kuhl (1820), but was then reclassified as a subspecies, *P. molurus bivittatus*, 100 years later. *Python molurus molurus* was differentiated as the other subspecies in the complex using subocular scales (McDiarmid, Campbell, & Touré, 1999). Most recently, *P. bivittatus* was again recognized as a distinct species with populations of *P. molurus* identified sympatrically (shared range) and possibly even syntopically (shared localities; Jacobs et al., 2009; Reynolds, Niemiller, & Revell, 2014; Schleip & O'Shea, 2010). The integrity of the two species and interbreeding avoidance in wild populations is thought to be maintained through resource partitioning of prey and microhabitat usage (O'Shea, 2007). Viable crosses, however, have been produced in captivity (Townson, 1980). Hybridization of the two species in the invasive range could affect climatic suitability and adaptation potential (as discussed previously) and also subsequent genetic analyses such as environmental DNA detection (Ryan et al., 2018; Wilcox et al., 2013). Here, we follow the most recent classification by Schleip and O'Shea (2010) and consider the Burmese python (*P. bivittatus*) and Indian python (*P. molurus*) as distinct species. To date, the GEE population has been morphologically identified as *Python bivittatus* throughout the invasive range.

A previous report of the invasive GEE population found one haplotype in cytochrome *b* (Cyt *b*) and two in the control region and used 10 cross‐species microsatellites developed by Jordan, Goodman, and Donnellan (2002) to conclude that the ENP population was not genetically structured (Collins, Freeman, & Snow, 2008). The sequence data and several locus‐specific and average genetic diversity values were not provided and therefore cannot be used for further comparison. Invasive Florida population‐specific microsatellite markers for *P. bivittatus* were subsequently isolated (*N* = 18) and combined with six cross‐species markers to identify 61% average expected heterozygosity (*H*_E_) and 2–6 alleles per locus (*N*_A_; 3.7 average *N*_A_; Hunter & Hart, 2013). In comparison, higher levels of genetic diversity (*N*_A_ = 10.88) were identified for *P. bivittatus* in the native range using eight microsatellites (Duan et al., 2017).

Our goal was to more thoroughly characterize the *P. bivittatus* populations in Florida to inform research and management strategies. We compared two mitochondrial DNA (mtDNA) genes with population‐specific nuclear microsatellite markers to investigate diversity, relatedness, effective population size, population structure, and introduction dynamics of *P. bivittatus* captured in Florida (Hunter & Hart, 2013). We further assessed phylogeographic structure and haplotype relationships and compared them with published sequences in an effort to assess the genetic origin and species composition of introduced pythons in Florida.

## 2 METHODS

### 2.1 Sample collection and DNA extraction

**Figure 1:**
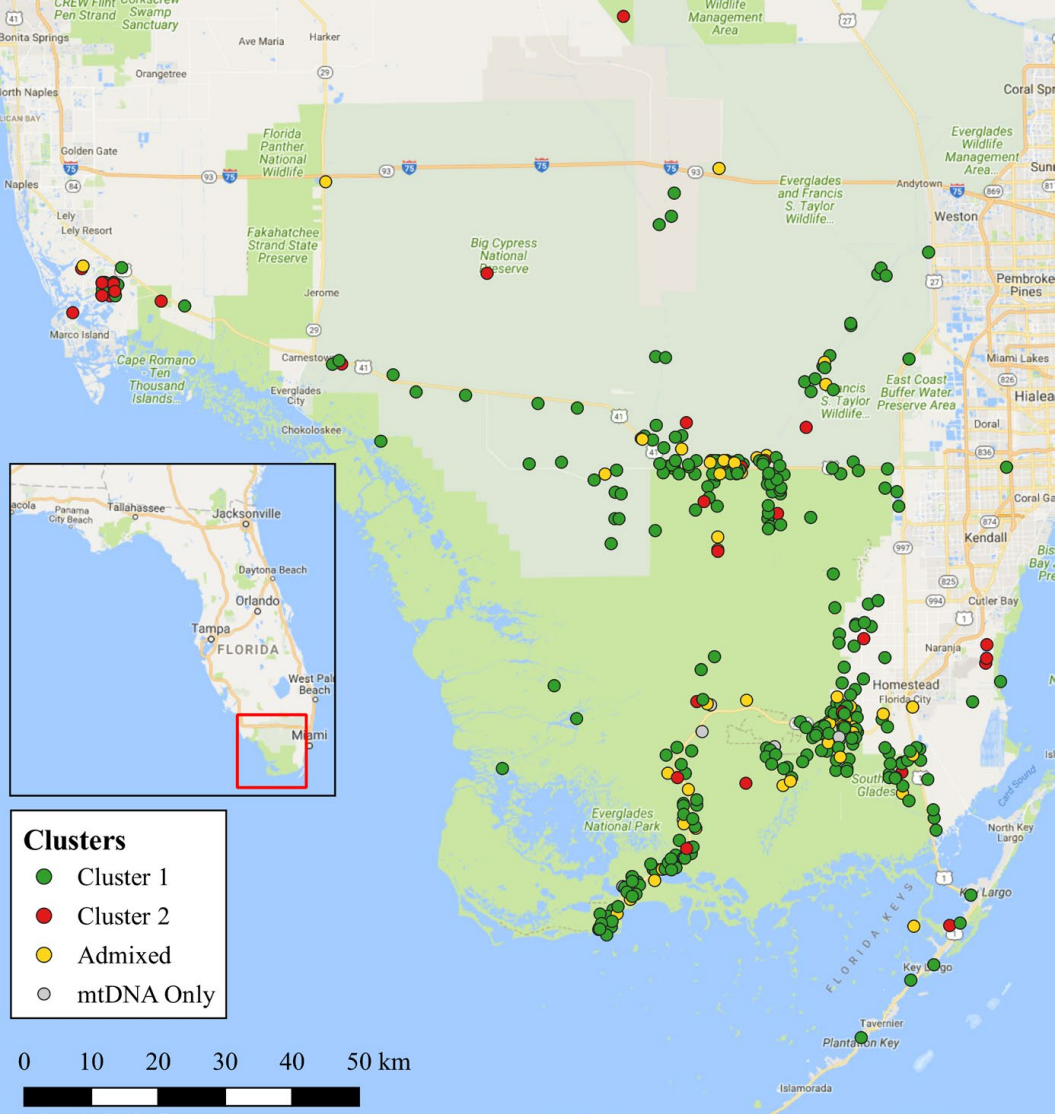
Map indicating python sample locations in southern Florida, USA. The nuclear Bayesian clustering assignments are shown in color. The samples yielding only mitochondrial DNA (mtDNA) sequences are in gray. Overlapping sample points have been offset to increase resolution [Colour figure can be viewed at http://wileyonlinelibrary.com]

The molecular analyses were conducted using tail tissue obtained from *P. bivittatus* samples collected January 2001 to September 2012. Samples originated in southern Florida from Everglades National Park, Collier County (including Big Cypress National Preserve), southeastern Miami‐Dade County, and the Florida Keys (Figure 1). Burmese pythons were identified by the presence of a subocular scale just below the eye, which differentiates them from *P. molurus,* which possess supralabial scales that extend from the lip to the bottom of the eye (O'Shea, 2007). All tissues were stored at −20°C. DNA was extracted using QIAGEN DNeasy kits (Valencia, CA) or plate isolation protocols (Whitlock, Hipperson, Mannarelli, & Burke, 2008). DNA was quantified by nanophotometer (Implen, Munchen, Germany) and diluted to 10 ng/μl.

### 2.2 Microsatellite analysis

#### 2.2.1 Microsatellite DNA analysis

To address fine‐scale genetic diversity and population structure in the invasive population, 18 population‐specific microsatellites were developed through next‐generation sequencing and incorporated with six cross‐species loci (Jordan et al., 2002) into eight multiplexes to reduce laboratory effort (Hunter & Hart, 2013). Of these markers, two loci (*MS16* and *MS22*) did not produce consistent scores and were excluded here. To optimize previously published annealing multiplex (MP) temperatures, *Pmb‐U21* was reassigned to MP1 and *MS09* was reassigned to MP9. Annealing temperatures and PCR parameters followed Hunter and Hart (2013), except for an annealing temperature of 57°C in MP4. All PCR products were analyzed on an ABI 3130xl (Applied Biosystems, Foster City, CA). Fragment data were scored using genemarker v. 1.97 (Soft Genetics, State College, PA). The majority of individual genotypes (*N* = 389) included all 22 loci, with a small percentage of samples missing ≤ seven loci.

#### 2.2.2 Microsatellite statistical analysis

micro‐checker (Van Oosterhout, Hutchinson, Wills, & Shipley, 2004) was used to identify loci with evidence of null alleles. genecap (Wilberg & Dreher, 2004) calculated the probability of identity (*P*_*(ID)*_), which is the probability that two individuals drawn at random from a population will have the same genotype at the assessed loci (Paetkau & Strobeck, 1994) and sibling probability of identity (*P*_*(ID)sib*_), a related, more conservative statistic for calculating *P*_*(ID)*_ among siblings (Evett & Weir, 1998). The program additionally searched for duplicate genotypes.

The program structure 2.3.4 (Pritchard, Stephens, & Donnelly, 2000) was used to identify the genetic relationships and population structure of the southern Florida population. structure, a model‐based clustering algorithm, infers population structure by probabilistically assigning individuals, without a priori geographic or ancestral knowledge, to a specific number (*K*) of clusters (presumably populations). In determining the number of clusters, the algorithm attempts to minimize deviations from Hardy–Weinberg equilibrium (HWE).

**Figure 2:**
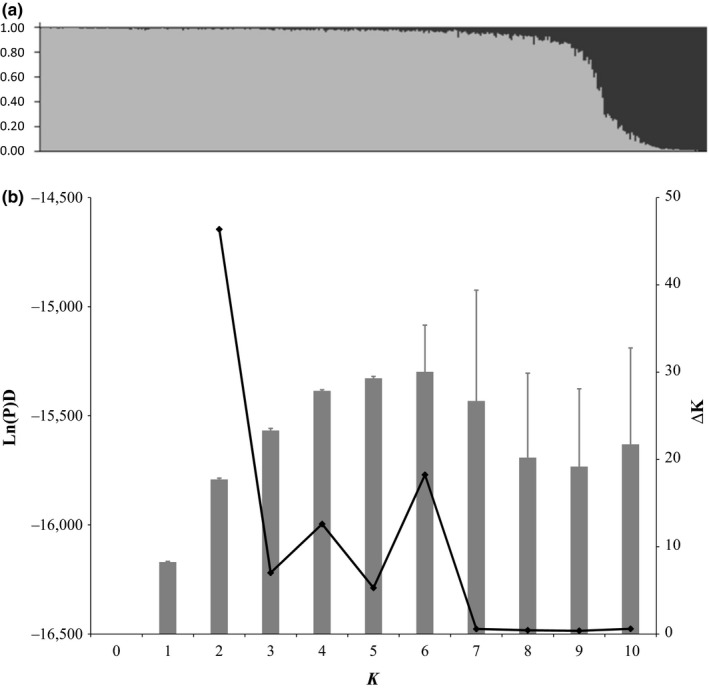
(a) Results of Bayesian clustering analysis (*K *=* *2) using 22 microsatellite loci with 389 python genotypes in structure 2.4.3 (cluster 1, gray; cluster 2, black. (b) The proportion of membership for *K *=* *2 was supported by the mean log likelihood (Ln P(D); denoted by bars) and Δ*K* (diamonds) versus *K*

Simulations were conducted using the correlated allele frequency model and admixture model, which assumes that individuals could have some proportion of membership (*q*) from each of *K* clusters. Multiple Markov chains can delineate differences within populations; therefore, 20 parallel chains were analyzed for *K *=* *1–11, with a run length of 200,000 Markov chain Monte Carlo repetitions, following a burn‐in period of 50,000 iterations. The most probable number of groups, *K*, was assessed using the mean log likelihood (Ln *P*(*D*)) and by calculating ∆*K*, an ad hoc quantity related to the change in posterior probabilities between runs of different *K* values (Evanno, Regnaut, & Goudet, 2005), in structure harvester (Figure 2; Cristescu, Sherwin, Handasyde, Cahill, & Cooper, 2010). Individual assignment success was recorded as the highest likelihood of assignment (*q*), and the percentage of individuals in a cluster with *q *≥* *0.90 was calculated. geneclass was used to detect first‐generation migrants born in a population other than the one in which they were sampled without *a priori* population categorization (Piry et al., 2004). We used the Paetkau, Calvert, Stirling, and Strobeck's (1995) simulation algorithm and *L*_*h*_ to assess the likelihood of finding individuals in the population in which they were sampled, which is most appropriate when all potential source populations have not been sampled. Migrant detection was assessed using the critical value (0.01; Paetkau, Slade, Burden, & Estoup, 2004).

The following statistical tests were conducted for the population as a whole and to assess the accuracy of the structure‐identified groups. The genetic diversity was estimated by the *H*_E_ and observed heterozygosity (*H*_O_), information index (*I*), *N*_A_, average effective number of alleles (*E*_A_), and private alleles (*P*_A_) using genalex 6.501 (Table 1, Supporting Information Table S1; Peakall & Smouse, 2006). Departures from the expected genotypic frequencies in HWE were tested using the Markov chain method, and linkage disequilibrium expectations were tested using the randomization method of Raymond and Rousset (1995) for all pairs of loci within collections to test for the presence of admixture in genepop 4.0 (dememorization, 1,000; batches, 100; iterations per batch, 1,000; Raymond & Rousset, 1995). Sequential Bonferroni adjustments (Rice, 1989) were used to determine significance for these tests.

**Table 1:** Summary statistics of the 22 polymorphic microsatellite loci for the *P. bivittatus* invasive population grouped by structure clusters. Number of individuals (*N*), average number of alleles (*N*_A_), effective number of alleles (*E*_A_), information index (*I*), observed heterozygosity (*H*_O_), expected heterozygosity (*H*_E_), and private alleles (*P*_A_). Individual locus information is provided in Supporting Information Table S1

Locus	*N*	*N*_A_	*E*_A_	*I*	*H*_O_	*H*_E_	*P*_A_
Cluster 1	263.59	3.18	2.53	0.96	0.58	0.59	1
Cluster 2	36.91	4.95	3.05	1.24	0.68	0.66	19
Admixed	49.23	4.55	2.63	1.06	0.58	0.61	10
Overall	349.73	5.50	2.63	1.05	0.59	0.60	20

To assess genetic differentiation of the clusters identified by structure, genalex 6.501 was used to calculate *F*_ST_ and *R*_ST_ via analysis of molecular variance (AMOVA) within and among clusters and individuals with 9,999 permutations. The statistical significance of the correlation between genetic and geographic distance matrices, or isolation by distance, was assessed with a Mantel randomization test performed with genalex 6.5 with 999 permutations comparing pairwise genetic distance (in meters; Paetkau & Strobeck, 1994). The genetic groupings were assessed by the LDne software (Waples & Do, 2008) to estimate effective population sizes (*N*_E_) using the linkage disequilibrium (LD) method at the three lowest allele frequency levels (0.01, 0.02, and 0.05), with 95% confidence intervals (CI) following the bias‐corrected method of Waples (2006). The single point estimate method removes the downward bias associated with the true *N*_E_ being greater than the sample size used to estimate it (Waples, 2006).

We used bottleneck 1.2.02 to evaluate heterozygote excess of populations under the sign test, one‐tailed Wilcoxon's signed‐rank test for mutation‐drift equilibrium, and the allele frequency distribution test (Table 2; Piry, Luikart, & Cornuet, 1999). The Garza–Williamson index and modified index were calculated in Arlequin 3.5 (Excoffier & Lischer, 2010). The Garza–Williamson index is the mean ratio of the number of alleles at a given locus to the range in allele size (*M*; Garza & Williamson, 2001). It is assumed that during a bottleneck event, the number of alleles decreases faster than the allelic range. A bottleneck is indicated with a critical value of *M *<* *0.68, and no reduction of effective population size is indicated at *M* >* *0.80.

**Table 2:** Microsatellite bottleneck analyses and effective population sizes (*N*_e_) for the three invasive python structure clusters . Two phase model (TPM) and stepwise mutation model (SMM)

Group	TPM		SMM		Mode‐shift	G‐W modified index	*N*_e_
	Sign test	Wilcoxon's test	Sign test	Wilcoxon's test			
Cluster1	0.00002	0.00000	0.00021	0.00000	Shifted mode	0.568	236.1
Cluster2	0.24128	0.14511	0.41772	0.48731	L‐shaped	0.766	44.3
Admixed	0.05562	0.01506	0.41838	0.23139	L‐shaped	0.834	32.4
Total	0.16001	0.62488	0.03070	0.97692	L‐shaped	0.723	315.1

genalex 6.501 was used to calculate *F*_IS_, which is close to zero when the population is undergoing random mating. Mean relatedness values (*r*_*xy*_) were computed for all pairwise relationships via ML‐RELATE (Kalinowski, Wagner, & Taper, 2006). Relatedness and individual inbreeding coefficients (*F*_*x*_) were estimated for all pairwise relationships via COANCESTRY (Wang, 2011). From mean relatedness values, latent coancestry (Ө_*xy*_) was calculated following Lynch and Ritland (1999), where *r*_*xy*_ = 2Ө_*xy*_. To better understand the capacity to increase genetic diversity in the population, relatedness was also estimated specifically for eight collected hatchlings from a single nest (P0003, P0006, P0008, P0009, P0016, P0020, P0021, P0024) to test for multiple paternity. Where not specified previously, the default parameters were used in the previous analyses.

### 2.3 Mitochondrial analysis

#### 2.3.1 Mitochondrial DNA analysis

Mitochondrial DNA variation was assayed at two protein‐coding loci: Cyt *b* (Rawlings, 2001) and cytochrome *c* oxidase I (CO1; Folmer, Black, Hoeh, Lutz, & Vrijenhoek, 1994). The PCR conditions were as follows: 10 ng DNA, 1× PCR buffer (10 mm Tris‐HCl, pH 8.3, 50 mm KCl, 0.001% gelatin; Sigma‐Aldrich, Inc., St. Louis, MO), 0.8 mm dNTP, 3 mm MgCl_2_, 0.24 μm of each primer, 0.04 units of Sigma Jump Start *Taq* DNA polymerase. PCR cycling profile: 5 min at 94°C; then 35 cycles of 1 min at 94°C, 1 min at 55°C, and 1 min at 72°C; then 10 min at 72°C. Amplified products were purified using ExoSap‐IT (Affymetrix, Santa Clara, CA) for PCR cleanup. DNA sequencing was accomplished with the BigDye terminator protocol (Applied Biosystems, Foster City, CA).

#### 2.3.2 Mitochondrial statistical analysis

Sequences were trimmed to those published in GenBank, checked for quality scores, and aligned in geneious 5.4.6 (Drummond et al., 2011). Representatives from each haplotype and any ambiguous sequences were sequenced in both directions to ensure the accuracy of nucleotide designations. We calculated summary statistics for the mtDNA by assessing nucleotide diversity (*π*), haplotype diversity (*h*), sequence diversity (*k*), and the standard neutrality test, Tajima's *D*, using DnaSP v5.0 (Table 3, Supporting Information Tables S2 and S3; Librado & Rozas, 2009). To assess the mtDNA and nuclear data collectively, the individuals containing both concatenated mtDNA sequences and microsatellite genotypes were assessed together (*N* = 293), while the concatenated mtDNA matrix only included those individuals sequenced at both loci (*n* = 399; Table 3). Using the microsatellite‐defined structure populations described below (cluster 1, cluster 2, or admixed), we calculated pairwise Φ_ST_ (10,000 permutations; *p* value <0.05 significant; Table 4) and exact tests of population differentiation (100,000 Markov chain steps; 10,000 dememorization steps; *p* value <0.05 significant) using Arlequin 3.5 (Excoffier & Lischer, 2010).

**Table 3:** Summary statistics for the invasive Florida python concatenated cytochrome *b* and cytochrome oxidase 1 sequences (1,397 bps; *N* = 399) for the *P. bivittatus* (Pb) and *P. molurus* (Pm) invasive haplotypes (H01 to H06). Within the sample groups, *N* represents the number of sequences; *S*, number of polymorphic sites; *H*, number of haplotypes; *h,* haplotype diversity; *π*, nucleotide diversity; *k*, average number of nucleotide differences; TD, Tajima's D, *significant (*p *≤* *0.05)

Sample groups	*N*	*S*	*H*	*h*	*π*	*k*	TD	Pb‐FL‐H01	Pb‐FL‐H02	Pb‐FL‐H03	Pb‐FL‐H04	Pm‐FL‐H05	Pb‐FL‐H06
Cluster 1	228	1	2	0.009	0.000	0.009	−0.938	227	0	0	1	0	0
Cluster 2	27	64	4	0.53	0.014	19.67	0.638	18	2	0	0	5	2
Admixed	38	61	3	0.104	0.002	3.260	−2.810*	36	0	1	0	1	0
Clusters 1, 2, admixed	293	65	6	0.080	0.002	2.535	−2.247*	281	2	1	1	6	2
Total sequenced	399	65	6	0.097	0.002	3.405	−1.918*	379	5	1	1	11	2

*Note*

Sequences are grouped by structure clusters obtained through nuclear microsatellite genotypes (*N* = 293) and sequences (*N* = 399). Note that 106 samples did not contain data for both marker types.

**Table 4:** Concatenated sequences grouped by structure clusters. Pairwise Φ_ST_ values below the diagonal and exact tests of population differentiation above the diagonal

	Cluster 1	Cluster 2	Admixed
Cluster 1	—	0.000*	0.054
Cluster 2	0.514*	—	0.002*
Admixed	0.074*	0.115*	—

*Note*

An asterisk (*) denotes significance at *p *<* *0.05.

Invasive samples were compared to GenBank and BOLD published sequences with similar length and quality (see Table 5 for sequence name abbreviations, references, and submission details). Complete mitochondrial DNA genomes were recently published for *P. molurus* (Dubey, Meganathan, & Haque, 2012) and *P. bivittatus* (Liu, Zhang, & Cao, 2013), accompanied by direct submissions of mtDNA sequences in GenBank. Slowinski and Lawson (2002) previously addressed phylogenies of 42 snake species using Cyt *b* and C‐mos genes; however, the *P. molurus* sequence did not include voucher or origin of sample information. The CO1 sequences published in BOLD contained two *P. molurus* samples with voucher specimens. We selected the longer sequence originating from a sample in a forested area in Maharashtra state in western India to avoid trimming our alignment (Sequence ID: ISDB081‐13.COI‐5P). The second sequence (ISDB016‐11.COI‐5P) was from a snake housed in a zoo in the same state and differed by four base pairs (bps) from ISDB081‐13. *Python regius* (Dong & Kumazawa, 2005) was included as the basal member of the python genus (Reynolds et al., 2014).

**Table 5:** References and GenBank accession number or BOLD sequence ID (http://www.boldsystems.org) for the published haplotypes used in the study

Name	Acc No/Seq ID	Reference	Direct submission	Country
Pb‐Ctb‐A	KF010492	Liu et al. (2013)	Yes	China
Pb‐Ctb‐B	KF293729	Liu et al. (2013)	Yes	China
Pm‐Ctb‐A	AY099983	Slowinski and Lawson (2002)		
Pm‐Ctb‐B	GQ225654	Dubey et al. (2009, 2002)	Yes	India
Pb‐CO1‐A	KF010492	Liu et al. (2013)	Yes	China
Pb‐CO1‐B	KF293729	Liu et al. (2013)	Yes	China
Pb‐CO1‐C	JX401103	You et al. (2013)		China
Pm‐CO1‐B	AB920233	Supikamolseni and Srikulnath (2014)	Yes	Thailand
Pm‐CO1‐A	ISDB081‐13	http://www.boldsystems.org	Yes	India
*Python regius*	AB177878	Dong and Kumazawa (2005)		

*Note*

The prefix indicates the most similar species (*P. bivittatus*, Pb; *P. molurus*, Pm), and the gene is identified as either cytochrome *b* (Ctb) or cytochrome oxidase 1 (CO1). Direct submission sequences deposited in the databases are not associated with a publication. Country of origin is indicated for the sample or authors.

Pairwise genetic distances were calculated using Tajima‐Nei between the invasive population samples and *P. molurus* sequences published in GenBank and the Barcode of Life Data (BOLD) system (http://www.barcodinglife.org; Ratnasingham & Hebert, 2007; Tajima & Nei, 1984) using MEGA7 (Kumar, Stecher, & Tamura, 2016; Supporting Information Tables S4 and S5). Polymorphic sites and the corresponding diagnostic sites were determined by the alignment of published haplotypes and haplotypes identified in this study (Supporting Information Tables S6 and S7). Genetic differentiation was tested with an analysis of molecular variance (AMOVA) in Arlequin 3.5, using models of DNA sequence evolution selected by the Akaike information criterion (AIC) and Bayesian information criterion (BIC) in MEGA7 (Darriba, Taboada, Doallo, & Posada, 2012; Excoffier & Lischer, 2010; Guindon & Gascuel, 2003). The T92 model (Tamura, 1992) was selected for CO1 sequences. The TN93 model (Tamura & Nei, 1993) was selected for Cyt *b* and concatenated (CO1 [*N* = 598] and Cyt *b* [*N* = 799]) analyses.

**Figure 3:**
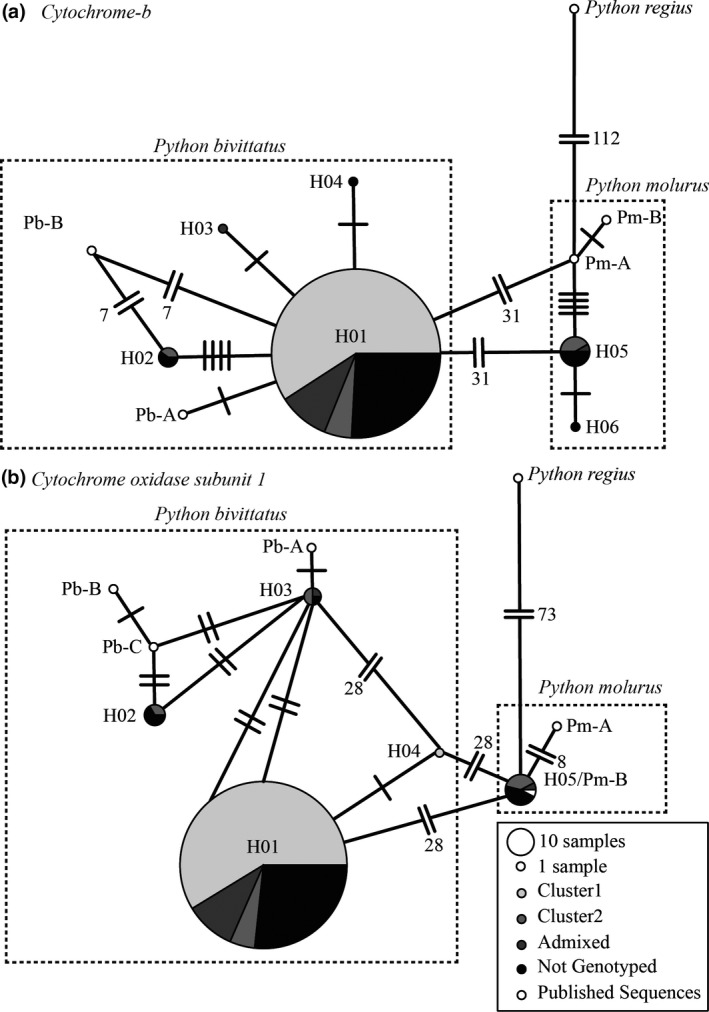
*Python bivittatus* (Pb) and *P. molurus* (Pm) (a) cytochrome *b* and (b) cytochrome oxidase 1 haplotype networks. Bayesian cluster assignment for invasive haplotypes (H01–H06) is denoted by gray shading. Published sequences’ (white circles) references are given in Table 5 and denoted by the species prefix. The area of each pie chart represents the number of haplotypes. Base pair discrepancies are given by the hash marks

We created haplotype networks using PopART (Leigh & Bryant, 2015) to assess the geographic distribution of mtDNA diversity and compare relationships between our samples and those previously published (Figure 3). Default minimum spanning network settings were used to generate a haplotype network with pie charts representative of the proportion of samples grouped by structure clusters. The number of base pair discrepancies between haplotypes is provided in parenthesis.

## 3 RESULTS

To summarize the results, 11 mtDNA haplotypes (GenBank Accession Number: MH357840‐50) were identified with high haplotype diversity in the invasive python samples corresponding to both *P. bivittatus* and *P. molurus*. Nuclear microsatellite markers detected lower diversity and *N*_*E*_ as compared to native range samples, likely related to founding and bottleneck effects. Bayesian clustering analyses identified two distinct nuclear groups and an admixed group with no correlation with geographic distribution. The *P. molurus* haplotypes were more predominantly classified in cluster 2.

### 3.1 Microsatellite DNA analysis

Only the *MS13* locus in cluster 1 indicated the evidence of null alleles due to homozygote excess (>0.05), but there was no evidence of stuttering, large allele dropout, or linkage disequilibrium. The loci produced an unbiased *P*_*(ID)*_ estimate of 5.63 E−15 and a *P*_*(ID)sib*_ estimate of 2.99 E‐07, indicating that unique individuals can be confidently identified across the region. The Bayesian structure Ln P(D) estimates indicated similar values and generally plateaued at *K *=* *4 clusters, while the structure harvester analysis according to Evanno et al. (2005) strongly supported *K *=* *2 clusters (Figure 2). The *K *=* *4 and next highest ∆*K* (*K *=* *6) were also investigated; however, the majority of the genotypes were “roughly symmetrically” assigned across the four or six populations, respectively, indicating that these values of *K* are not identifying real population structure (Supporting Information Figure S1; Pritchard et al., 2000). Therefore, as recommended, *K *=* *2 was selected and groups were assigned as follows: cluster 1 (*N* = 292), cluster 2 (*N* = 42), and a third, admixed group (*N* = 55), containing *q *≤* *90% assignment to the two clusters (Figure 2; Evanno et al., 2005; Pritchard et al., 2000).

Across the 389 samples, Hardy–Weinberg disequilibrium was found for *Pmb‐N14* and *Pmb‐Z26* (*p* ≤ 0.002). After the sequential Bonferroni adjustments, linkage disequilibrium was found for 39 of 231 (16.9%) comparisons, likely due to population substructure tested below. Separate analyses of the three groups identified in structure resulted in HWE for all loci and linkage equilibrium for cluster 1 and admixed. However, linkage disequilibrium was found in cluster 2 for two pairs of loci (*Pmb‐S19* and *Pmb‐R18*,* Pmb‐N14* & *Pmb‐K11*). This deviation may be due to inbreeding or cryptic subpopulation structure (i.e., Wahlund effect). geneclass detected 16 samples with a probability <0.01. These samples were all members of cluster 2 defined by structure.

*F*_*ST*_ values among the three structure‐defined clusters were low, but significant (*p* ≤ 0.017): cluster 1 versus 2 (0.029), cluster 1 versus admixed (0.004), and cluster 2 versus admixed (0.012). *R*_ST_ values were not significant, likely owing to the minimal time for mutations to occur. Low levels of nuclear diversity were found for all samples assessed together and grouped by clusters (Table 1). The AMOVA identified moderate variation both among the three structure clusters and within individuals (19.12% and 1.49%, respectively). In cluster 2, 19 private alleles were found in 33 samples, while the admixed group contained 10 private alleles in 14 samples distributed across the q‐values. Cluster 1 had a single private allele in five samples. Effective population sizes using the linkage disequilibrium method were similar for the three allele frequencies tested; therefore, the 0.01 frequencies are reported (Table 2). The Mantel test indicated no significant correlations between genetic and geographic distances (*p *=* *0.27).

Assessing the 389 samples together, the stepwise mutational model (SMM) of the sign test was significant (*p *=* *0.03). However, a normal “L”‐shaped allele distribution curve was obtained, indicating a larger proportion of alleles in the low‐frequency allele classes (Table 2). All bottleneck tests for cluster 1 were significant, and a bottleneck was also indicated with a Garza–Williamson modified index value below the critical value (*M *=* *0.568). Cluster 2 indicated a reduction of effective population size (*M *=* *0.766), but was not below the critical value threshold. The admixed group was significant under the Wilcoxon's TPM (*p *=* *0.015). Nonsignificant bottleneck test values may have been due to smaller sample sizes.

The inbreeding coefficient, *F*_IS_, was 0.194 (*p *=* *0.000) over the three groups, which indicates inbreeding and/or a founding effect on the population. Overall, the average number of alleles was 5.50 and *H*_E_ was 0.60 (Table 1). Relatedness levels (*r*_*xy*_ = 0.091) were between first (*r*_*xy*_ = 0.125) and second (*r*_*xy*_ = 0.0625) cousins on average, and inbreeding coefficients were also indicative of a cousin relationship. Across the structure cluster and all estimators, cluster 1 had the highest level of relatedness, cluster 2 had moderate levels, and admixed had the lowest level. Simulations using population allele frequencies estimated that 20% of the population was related, while observed values estimated that 24% of the samples were related. Analysis of the small number of collected hatchlings indicated that they were likely related at either the half‐sibling or full‐sibling levels.

### 3.2 Mitochondrial DNA analysis

Cytochrome *b* produced six novel haplotypes in 419 sequences across 799 bps with relatively high genetic distance (range 0.13%–4.30%) and number of polymorphic sites (*S* = 1–36 bps; Supporting Information Tables S4 and S6). Cytochrome oxidase 1 produced five haplotypes in 413 sequences across 585 bps with high genetic distances (0.30%–5.40%) and numbers of polymorphic sites (*S* = 1–31 bps; Supporting Information Tables S5 and S7). The invasive haplotypes split into two strongly divided groups at COI. The H01 sequences most closely associated with the published *P. bivittatus* mtDNA genomes (>99.14%; Liu et al., 2013) and H05 matching a published *P. molurus* sequence (Supikamolseni & Srikulnath, 2014, direct NCBI submission). The current published *P. bivittatus* and *P. molurus* sequences were ≥94.8% similar (Supporting Information Table S5).

The concatenated sequences produced six novel haplotypes in 399 snakes across 1,397 bps. The majority of samples were found to be a single haplotype (Pb‐FL‐H01; *N* = 379), with the five other haplotypes represented in lower proportions (Table 3). The Pm‐FL‐H05 haplotype was found in 11 samples associated with the *P. molurus* mitotype. No phylogeographic pattern was found in accordance with collection sites in southern Florida (Figure 1).

### 3.3 Mitochondrial DNA haplotypes partitioned by structure clusters

The structure cluster 1 contained only the Pb‐FL‐H01 haplotype in all but one sample, while cluster 2 and the admixed groups contained a mixture of haplotypes (Table 3). The majority of the haplotypes were each assigned to a single cluster (Table 3). Interestingly, although not selected as the correct grouping, many of the samples with *P. molurus* haplotypes were assigned to a single cluster in the *K *=* *4 plot (green; Supporting Information Figure S1). Diagnostic sites differentiating the two *Python* species were identified for the two loci: Cyt *b* (*N* = 27) and CO1 (*N* = 24; Supporting Information Tables S6 and S7). The highest differences between concatenated sequences, as measured by Φ_ST_ and exact test values, were between the cluster 1 and 2 structure groups (*p* < 0.05; Table 4). The mtDNA AMOVA values within and among the three structure groups resulted in relatively high variation (69.96% and 30.04%, respectively). The AMOVA identified variation levels of 48.60% and 51.40% within and among clusters 1 and 2, respectively.

### 3.4 Comparison with published sequences

The dominant Cyt *b* haplotype that we found in the invasive range matched all but one nucleotide to one of the published *P. bivittatus* mitochondrial genome sequences (Pb‐Ctb‐A; Supporting Information Table S4; Liu et al., 2013). The next highest frequency haplotype differed by ≥4.13% from Liu et al. (2013), but was only ≤0.76% different from the published *P. molurus* sequences (Dubey et al., 2012; Slowinski & Lawson, 2002). A similar pattern was found for the CO1 haplotypes with a dominant haplotype most closely resembling *P. bivittatus*. The second most dominant haplotype matched *P. molurus* or differed by 1.38% (Supporting Information Table S5). No subocular differentiation was found in the available photographs of the snakes containing *P. molurus* haplotypes. There was some disparity between the published sequences and associated species labels, which may relate to the lack of consensus in the nomenclature.

## 4 DISCUSSION

The invasive Burmese python population in Florida appears to be derived from multiple genetic sources with strongly divergent mitotypes corresponding to species‐level differentiation. The Cyt *b* genetic distance (4.3%) was larger than the distance found in the most recent taxonomic assessment that separated *P. bivittatus* and *P. molurus* into species (2.9%; Reynolds et al., 2014). The CO1 genetic distance (5.4%) was also greater than the distances for the two species published in BOLD (4.1%). In the literature, CO1 nucleotide diversity values lower than 4.1% have been used as a minimum threshold to distinguish intraspecific variation from interspecific divergence (Gomes, Pessali, Sales, Pompeu, & Carvalho, 2015; Ratnasingham & Hebert, 2013). In contrast to the strong mitochondrial differentiation signals, minimal divergence was detected between the three Bayesian clusters at nuclear microsatellites (*F*_ST_ ≤ 0.029).

In vertebrates, cytonuclear discordance is indicated by conflicting signals between mtDNA and nuclear genetic diversity. The nonrecombining mitochondrial genome sequence remains as a distinct *cytotype* in an admixed clade until, over time, only either the dominant (parent) or introgressed cytotype is retained (see Seehausen, 2004). On the contrary, admixed nuclear genotypes will continue to recombine with the dominant alleles until introgressed. During the intermediate phase, nuclear genome hybrids and both cytotypes can be detected. Cytonuclear discordance can be caused by hybridization, incomplete lineage sorting, direct balancing selection, indirect selection, or pseudogenes (Grobler, Jones, Johnson, Neves, & Hallerman, 2011; Thielsch, Knell, Mohammadyari, Petrusek, & Schwenk, 2017). In our data, incomplete lineage sorting is unlikely due to the high sequence divergence among the samples and rapid progression of lineage sorting in mitochondrial loci (Funk & Omland, 2003). Similarly, no evidence for indirect selection or pseudogenes causing cytonuclear discordance was observed here, given the haplotype sequences and divergence levels. It is possible that we detected direct balancing selection of rare ancestral mitochondrial lineages that favor specific environmental conditions; however, the role that mtDNA plays in natural selection is not fully understood (Funk & Omland, 2003). Most likely, the cytonuclear divergence we detected is the result of hybridization between snakes contributing mitochondrial genome sequences from both species.

Past hybridization of *P. molurus* and *P. bivittatus* may have led to the identified cytonuclear discordance in the invasive population. The nuclear *F*_ST_ values ≤0.029 suggest significant introgression of the nuclear genomes and a population‐level, as opposed to a species‐level, divergence. However, residual *P. molurus* nuclear genomic material may be contributing to the cluster 2 and admixed genotypes. For instance, in cluster 2, 16 first‐generation migrants were detected, and 19 private alleles were found in 79% of the individuals (with 10 individuals identified in both analyses). In natural systems, the sigmoid shape of the structure plot (Figure 2) can be interpreted as hybridization followed by selection against the hybrid alleles or segregating variation. However, as this population was likely released from the pet trade, the admixed group may indicate separate introductions of two relatively similar gene pools that are now interbreeding over a few generations.

The taxonomic uncertainty regarding species boundaries in the genus *Python* complicates our understanding of the precise mechanism responsible for the cytonuclear discordance. Introgression of the diverse lineages could occur through interbreeding (a) in the native range through sympatric associations or secondary contact (Seehausen, 2004), (b) during secondary contact in captivity, or (c) after release into the invasive range, possibly in part due to unidirectional hybridization or unbalanced sex ratios in hybrid generations (Firmat, Alibert, Losseau, Baroiller, & Schliewen, 2013). The support for scenario 3 is limited, given the low diversification in the nuclear genome suggestive of admixture over numerous generations. More extensive native range phylogeographic sampling of mtDNA and nuclear DNA loci is necessary to confirm whether the cytonuclear discordance observed in Florida is present in native populations or occurred after capture. Limited introgression of *P. molurus* followed by backcrossing to *P. bivittatus* may have occurred in the large, widely distributed commercial trade populations. Alternatively, intraspecific genetic divergence in the *P. bivittatus* native range may have contributed to the three nuclear groups found in China (*F*_ST_ = 0.11 overall), although assessment of mitochondrial DNA is needed to determine whether divergent mitotypes are also present (Duan et al., 2017).

Field observations in the native range indicate that the two species utilize distinct habitats with some overlapping ranges. *Python bivittatus* prefers riverine forests and flooded grasslands, while *P. molurus* occupies dry, sandy, and woodland areas (Schleip & O'Shea, 2010). Hybridization of the two species could allow for improved acclimatization and adaptability to abiotic stressors or climate change and result in broader or more rapid distributions of the invasive population (Hoffmann & Sgrò, 2011; Mazzotti et al., 2011; Rodda, Jarnevich, & Reed, 2009). Currently, pythons occupy both wetlands in Everglades National Park and drier, sandy pinelands with interspersed wetlands in western Collier County. However, evidence of a panmictic population was found with no temporal or phylogeographic pattern across the sampled range. This is not surprising, given that invasive pythons are known to disperse long distances (Hart et al., 2015).

A bottleneck and/or founding event was indicated 0.2–4 *N*_E_ generations ago with a ≥90% reduction in population size (Williamson‐Natesan, 2005). Given that Burmese pythons require two to five years to reach sexual maturity (Willson, Snow, Reed, & Dorcas, 2014), the population would have undergone approximately four to 10 generations after being founded in the mid‐1980s (Willson et al., 2011). The detection of a bottleneck of less than four generations ago may indicate a secondary bottleneck due to novel environmental conditions such as cold‐induced mortality (Mazzotti et al., 2011). Alternatively, a lag in the generation times or in the population growth rates may have occurred shortly after the population's founding, possibly due to low propagule pressure (Fujisaki et al., 2010). Reproduction was not documented until the first wild hatchlings were found in the mid‐1990s (Meshaka, Loftus, & Steiner, 2000), although detection of pythons has remained low until recently (Hunter et al., 2015; Reed et al., 2011). Parthenogenesis has also been identified in the species, which may allow for population expansion even at low densities and would contribute to reduced genetic diversity (Groot, Bruins, & Breeuwer, 2003).

In comparison with our findings, on average, native range Burmese pythons had nearly twice the number of alleles and higher average heterozygosities, with the exception of the Yunnan population, which had similar allelic values (*N*_A_ = 5; Duan et al., 2017). In the invasive population, effective population sizes were relatively low, supporting the hypothesis that the population was established by a small number of founders and/or closely related individuals (Willson et al., 2011). Monitoring of the effective population size could help to identify changes in the census size as genetic mutations occur and accumulate, especially in order to assess effective control efforts (Hauser, Adcock, Smith, Bernal Ramãrez, & Carvalho, 2002; Hui & Burt, 2015). More accurate effective population size estimates with lower variance can be calculated with genetic data collected over multiple generations (Hui & Burt, 2015).

While the genetic diversity in the invasive Burmese python population is lower than that found in the native range, it is likely to increase in the large, rapidly growing invasive population, especially if additional animals are released. Multiple paternity was identified in the invasive population which could also contribute to accelerated increases in diversity. Of note, the genetic confirmation of multiple breeding events by different sires lends support to the Judas control technique in which radio‐tagged snakes are used to reveal the location of conspecifics during breeding (Smith et al., 2016). Over time, as the population expands, some genotypes may become isolated or fixed, adapting to certain habitats and creating more population structure.

Recently, eDNA has become an important tool to estimate occurrence and detection probabilities and track the invasion front of the Burmese python populations (Hunter et al., 2015; Piaggio et al., 2013). Highly divergent mtDNA sequences could lead to mispriming of eDNA primers or probes, resulting in false negatives, along with potentially lower detection and occurrence estimates (Wilcox, Carim, McKelvey, Young, & Schwartz, 2015; Wilcox et al., 2013). Deep sampling is necessary to detect intraspecific variation found at low frequencies in the population.

The limited number of well‐documented, high‐quality published sequences hinders our ability to investigate *P. bivittatus* and *P. molurus* species boundaries. Morphological voucher specimens and broader phylogeographic sampling throughout the native range, including sympatric areas, could improve taxonomic uncertainty. Further, genomic‐level assessment and transcriptomic studies could address fine‐scale population structure and the Burmese python's adaptation to the novel environment (Castoe et al., 2011; Rodda et al., 2009; Wall et al., 2011). Findings from these endeavors could facilitate management in a variety of ways, including the development of effective monitoring tools (e.g., eDNA assays) and more accurate range expansion predictions.

## CONFLICT OF INTEREST

None declared.

## AUTHOR CONTRIBUTIONS

MEH designed the research, performed the research, analyzed the data, and wrote the paper. KMH and RWS designed the research, collected samples, and wrote the paper. BJS collected samples and wrote the paper. MCD performed the research, analyzed the data, and wrote the paper. NAJ and JSSB analyzed the data and wrote the paper.

## DATA ACCESSIBILITY

The data presented in this manuscript are available from https://doi.org/10.5066/f7hh6hkj.
