# Supplementary material for: Cytonuclear discordance in the Florida Everglades invasive Burmese python (Python bivittatus) population reveals possible hybridization with the Indian python (P. molurus)
Source: Ecol Evol. 2018 Aug 19;8(17):9034–47. doi: 10.1002/ece3.4423 (PMC6157680; doi:10.1002/ece3.4423)
Supplement: Supplementary file 1 [file ECE3-8-9034-s001.docx]

**Supplemental Tables and Figures**

**Table S1.** Characteristics of 22 polymorphic microsatellite loci for the *P. bivittatus* invasive population and average values for the Structure clusters. Number of individuals (N), average number of alleles (N_A_), effective number of alleles (E_A_), information index (I), observed heterozygosity (H_O_), and expected heterozygosity (H_E_).

| **Locus** | **N** | **N_A_** | **E_A_** | **I** | **H_O_** | **H_E_** |
| --- | --- | --- | --- | --- | --- | --- |
| **A01** | 331 | 2 | 1.99 | 0.69 | 0.46 | 0.5 |
| **B02** | 369 | 3 | 2.43 | 0.99 | 0.54 | 0.59 |
| **D04** | 348 | 6 | 2.52 | 1.08 | 0.6 | 0.6 |
| **F06** | 366 | 4 | 1.76 | 0.69 | 0.39 | 0.43 |
| **G07** | 296 | 4 | 2.76 | 1.08 | 0.63 | 0.64 |
| **J10** | 382 | 4 | 2.02 | 0.76 | 0.48 | 0.51 |
| **K11** | 358 | 5 | 2.91 | 1.13 | 0.66 | 0.66 |
| **L12** | 363 | 9 | 2.49 | 1.07 | 0.61 | 0.6 |
| **MS10** | 323 | 7 | 2.3 | 1 | 0.64 | 0.57 |
| **MS11** | 353 | 9 | 2.59 | 1.14 | 0.61 | 0.62 |
| **MS13** | 353 | 6 | 2.39 | 1.08 | 0.53 | 0.58 |
| **MS9** | 314 | 7 | 2.94 | 1.26 | 0.6 | 0.66 |
| **N14** | 353 | 6 | 2.85 | 1.19 | 0.68 | 0.65 |
| **O15** | 372 | 4 | 2.86 | 1.11 | 0.64 | 0.65 |
| **Q17** | 352 | 6 | 4.13 | 1.46 | 0.73 | 0.76 |
| **R18** | 381 | 8 | 3.01 | 1.19 | 0.67 | 0.67 |
| **S19** | 366 | 6 | 2.15 | 0.86 | 0.5 | 0.54 |
| **T20** | 301 | 3 | 1.85 | 0.73 | 0.53 | 0.46 |
| **U21** | 310 | 6 | 2.71 | 1.07 | 0.59 | 0.63 |
| **V22** | 384 | 6 | 2.16 | 0.87 | 0.55 | 0.54 |
| **W23** | 336 | 4 | 3.91 | 1.38 | 0.73 | 0.75 |
| **Z26** | 383 | 6 | 3.06 | 1.18 | 0.64 | 0.67 |
| **Overall Average** | 349.73 | 5.50 | 2.63 | 1.05 | 0.59 | 0.60 |
|  |  |  |  |  |  |  |
| **Cluster 1 Average** | 263.59 | 3.18 | 2.53 | 0.96 | 0.58 | 0.59 |
| **Cluster 2 Average** | 49.23 | 4.55 | 2.63 | 1.06 | 0.58 | 0.61 |
| **Admixed Average** | 36.91 | 4.95 | 3.05 | 1.24 | 0.68 | 0.66 |

**Table S2.** Summary statistics for invasive Florida python sample groups for Cytochrome b sequences (799 bps). Sequences are grouped by nuclear microsatellite Structure Clusters (1, 2, and admixed; N=305 genotypes) and the total number of obtained sequences (N=419). Note that 114 samples did not contain data for both marker types. Statistics reported for each sample group: number of sequences (N), number of polymorphic sites (S), number of haplotypes (H)*,* haplotype diversity (*h*), nucleotide diversity (π), average number of nucleotide differences (k), and Tajima’s D (TD) (**P* ≤ 0.05 significance).

| Sample groups | N | S | H | *h* | π | k | TD | Pb-FL-Ctb01 | Pb-FL-Ctb02 | Pb-FL-Ctb03 | Pb-FL-Ctb04 | Pm-FL-Ctb05 | Pm-FL-Ctb06 |
| --- | --- | --- | --- | --- | --- | --- | --- | --- | --- | --- | --- | --- | --- |
| Cluster 1 | 236 | 0 | 1 | 0 | 0 | - | - | 236 | 0 | 0 | 0 | 0 | 0 |
| Cluster 2 | 41 | 32 | 3 | 0.096 | 0.0020 | 1.561 | -2.727* | 39 | 0 | 1 | 0 | 1 | 0 |
| Admixed | 28 | 33 | 3 | 0.415 | 0.0124 | 9.902 | 0.492 | 21 | 2 | 0 | 0 | 5 | 0 |
| Cluster 1, 2, Admix | 305 | 34 | 4 | 0.058 | 0.0016 | 1.258 | -2.147* | 296 | 2 | 1 | 0 | 6 | 0 |
| Total Sequenced | 419 | 36 | 6 | 0.092 | 0.0025 | 1.975 | -1.761* | 399 | 5 | 1 | 1 | 12 | 1 |

**Table S3.** Summary statistics for invasive Florida python sample groups for CO1 sequences (585 bps). Sequences were grouped by nuclear microsatellite Structure clusters (1, 2, and admixed) determined using commensurate nuclear microsatellite genotypes (N=298) for the total number of obtained sequences (N=413). Note that 115 samples did not contain data for both marker types. Statistics reported for each sample group: number of sequences (N), number of polymorphic sites (S), number of haplotypes (H)*,* haplotype diversity (*h*), nucleotide diversity (π), average number of nucleotide differences (k), and Tajima’s D (TD) (**P* ≤ 0.05 significance).

| Sample groups | N | S | H | *h* | π | k | TD | Pb-FL-CO1-01 | Pb-FL- CO1-02 | Pb-FL- CO1-03 | Pb-FL- CO1-04 | Pm-FL-CO1-05 |
| --- | --- | --- | --- | --- | --- | --- | --- | --- | --- | --- | --- | --- |
| Cluster 1 | 230 | 1 | 2 | 0.009 | 1E-05 | 0.0087 | -0.9375 | 229 | 0 | 0 | 1 | 0 |
| Cluster 2 | 40 | 29 | 3 | 0.099 | 0.0025 | 1.4974 | -2.6783* | 38 | 0 | 1 | 0 | 1 |
| Admixed | 28 | 31 | 4 | 0.516 | 0.0154 | 9.1958 | 0.56617 | 19 | 2 | 2 | 0 | 5 |
| Cluster 1, 2, Admix | 298 | 31 | 5 | 0.079 | 0.002 | 1.2066 | -2.1004* | 286 | 2 | 3 | 1 | 6 |
| Total Sequenced | 413 | 31 | 5 | 0.107 | 0.003 | 1.7376 | -1.7188 | 390 | 6 | 4 | 1 | 12 |

**Table S4.**  Genetic distance across 799 bps of Cytochrome *b* sequenced in this study **(**denoted FL; N**=**419**)** and published in Genbank (below the diagonal). Genbank published haplotypes are *Python bivittatus:* Pb-A (Liu *et al.* 2013 [Acc. No. KF010492], Complete genome, China), Pb-B (Liu *et al.* 2013, Complete genome, [Acc. No. KF293729], China). *P. molurus:* Pm-A **(**Slowinski and Lawson 2002 [Acc. No. AY099983]), Pm-B (Dubey *et al.* 2009 [Acc. No. GQ225654], India). *P. regius:* (Dong and Kumazawa 2005 [Acc. No. AB177878]). Standard error values are given above the diagonal.

| Haplotype | Pb-A | Pb-B | Pb-FL-H01 | Pb-FL-H02 | Pb-FL-H03 | Pb-FL-H04 | Pm-FL-H05 | Pm-FL-H06 | Pm-A | Pm-B | P.regius |
| --- | --- | --- | --- | --- | --- | --- | --- | --- | --- | --- | --- |
| Pb-A |  | 0.004 | 0.001 | 0.003 | 0.002 | 0.002 | 0.007 | 0.007 | 0.007 | 0.007 | 0.016 |
| Pb-B | 0.01 |  | 0.003 | 0.003 | 0.003 | 0.004 | 0.007 | 0.007 | 0.007 | 0.008 | 0.016 |
| Pb-FL-H01 | 0.001 | 0.009 |  | 0.002 | 0.001 | 0.001 | 0.007 | 0.007 | 0.007 | 0.007 | 0.016 |
| Pb-FL-H02 | 0.006 | 0.009 | 0.005 |  | 0.003 | 0.003 | 0.007 | 0.007 | 0.007 | 0.007 | 0.016 |
| Pb-FL-H03 | 0.003 | 0.010 | 0.001 | 0.006 |  | 0.002 | 0.007 | 0.007 | 0.007 | 0.007 | 0.016 |
| Pb-FL-H04 | 0.003 | 0.010 | 0.001 | 0.006 | 0.003 |  | 0.007 | 0.007 | 0.007 | 0.007 | 0.016 |
| Pm-FL-H05 | 0.042 | 0.043 | 0.040 | 0.042 | 0.042 | 0.042 |  | 0.001 | 0.002 | 0.003 | 0.016 |
| Pm-FL-H06 | 0.043 | 0.045 | 0.042 | 0.043 | 0.043 | 0.043 | 0.001 |  | 0.003 | 0.003 | 0.016 |
| Pm-A | 0.042 | 0.043 | 0.040 | 0.042 | 0.042 | 0.042 | 0.005 | 0.006 |  | 0.001 | 0.016 |
| Pm-B | 0.043 | 0.045 | 0.042 | 0.043 | 0.043 | 0.043 | 0.006 | 0.008 | 0.001 |  | 0.016 |
| P.regius | 0.169 | 0.173 | 0.167 | 0.169 | 0.169 | 0.169 | 0.164 | 0.164 | 0.159 | 0.161 |  |

**Table S5.**  Genetic distance across 585 bps of cytochrome oxidase 1 (CO1) sequenced in this study **(**denoted FL; N**=**413) and published in GenBank (below the diagonal). Genbank published haplotypes are *Python bivittatus:* Pb-CO1-A ( [Acc. No. KF010492], China), Pb-CO1-B ( Liu et al. (2013) [Acc. No. KF293729], China), Pb-CO1-C ([Acc. No. JX401103], China). *P. molurus:* Pm-CO1-B (Supikamolseni and Srikulnath 2014 [Acc. No. AB920233], Thailand). The *P. molurus* BOLD published sequence is Pm-CO1-A (BOLD sequence ID: ISDB081-13). *P. regius:* (Dong and Kumazawa 2005 [Acc. No. AB177878]). Standard error values are given above the diagonal.

| Haplotype | Pb-A | Pb-B | Pb-C | Pb-FL-H01 | Pb-FL-H02 | Pb-FL-H03 | Pb-FL-H04 | Pm-FL-H05 | Pm-A | Pm-B | P.regius |
| --- | --- | --- | --- | --- | --- | --- | --- | --- | --- | --- | --- |
| Pb-A |  | 0.003 | 0.003 | 0.003 | 0.003 | 0.002 | 0.003 | 0.01 | 0.01 | 0.01 | 0.022 |
| Pb-B | 0.007 |  | 0.002 | 0.004 | 0.003 | 0.003 | 0.004 | 0.01 | 0.011 | 0.01 | 0.023 |
| Pb-C | 0.005 | 0.002 |  | 0.003 | 0.002 | 0.002 | 0.003 | 0.01 | 0.01 | 0.01 | 0.022 |
| Pb-FL-H01 | 0.005 | 0.009 | 0.007 |  | 0.003 | 0.002 | 0.002 | 0.009 | 0.01 | 0.009 | 0.022 |
| Pb-FL-H02 | 0.005 | 0.005 | 0.003 | 0.007 |  | 0.002 | 0.003 | 0.01 | 0.01 | 0.01 | 0.022 |
| Pb-FL-H03 | 0.002 | 0.005 | 0.003 | 0.003 | 0.003 |  | 0.002 | 0.01 | 0.01 | 0.01 | 0.022 |
| Pb-FL-H04 | 0.005 | 0.009 | 0.007 | 0.002 | 0.007 | 0.003 |  | 0.009 | 0.01 | 0.009 | 0.022 |
| Pm-FL-H05 | 0.052 | 0.056 | 0.054 | 0.050 | 0.054 | 0.050 | 0.05 |  | 0.005 | 0 | 0.02 |
| Pm-A | 0.056 | 0.06 | 0.058 | 0.054 | 0.058 | 0.054 | 0.054 | 0.014 |  | 0.005 | 0.02 |
| Pm-B | 0.052 | 0.056 | 0.054 | 0.050 | 0.054 | 0.050 | 0.05 | 0 | 0.014 |  | 0.02 |
| P.regius | 0.164 | 0.169 | 0.167 | 0.162 | 0.167 | 0.162 | 0.162 | 0.14 | 0.144 | 0.14 |  |

**Table S6.** Cytochrome *b* (Ctb) sequence alignment of polymorphic sites (N=44) within 799 bps of *P. bivittatus* and *P. molurus* haplotypes. Invasive population haplotypes denoted FL-Ctb-H01 to -H06 with a Pb- or Pm-prefix determined by the most related published sequences. Published sequences are Pb-Ctb-A (Liu *et al.* 2013 [Genbank Acc. No. KF010492], China), Pb-Ctb-B (Liu *et al.* 2013 [Acc. No. KF293729], China), Pm-Ctb-A **(**Slowinski and Lawson 2002 [Acc. No. AY099983]), and Pm-Ctb-B (Dubey *et al.* 2010 [Acc. No. GQ225654.1], India). Vertical numbers represent polymorphic base pair positions within the alignment and dots indicate sequence homology with the Pb-Ctb-A haplotype. Diagnostic sites differentiating the two species (N=27) are underlined.

|  | Polymorphic sites |
| --- | --- |
|  | 11112223333334444444455555555666777777 |
| Haplotypes | 167705791781125890114466911334557113347899 |
|  | 45350394907493685573475839647584397022122069 |
| Pb-Ctb-A | ACTACAATTACTTGCTCCAGCTCCCTTAACAGTTGTCTGCACAT |
| Pb-Ctb-B | ..C.....C....A.GTT.....TT................... |
| Pb-FL-Ctb-H01 | ..C......................................... |
| Pb-FL-Ctb-H02 | ..C..........A.G.....C..................G... |
| Pb-FL-Ctb-H03 | ..CG........................................ |
| Pb-FL-Ctb-H04 | ..C...................................C..... |
| Pm-FL-Ctb-H05 | GTC.TGGCCGTCCATA..GAT.T..CCGGT..CCA.TC.T.T.C |
| Pm-FL-Ctb-H06 | GTC.TGGCCGTCCATA..GAT.T..CCGGT..CCACTC.T.T.C |
| Pm-Ctb-A | ..C.TGGCCGTCCATA..GAT.T..CCGGTGACCA.TC.T.T.C |
| Pm-Ctb-B | ..C.TGGCCGTCCATA..GAT.T..CCGGTGACCA.TC.T.TCC |

**Table S7.** Cytochrome oxidase 1 (CO1) sequence alignment of polymorphic sites (N=39) within 585 bps of *P. bivittatus* and *P. molurus* haplotypes. Invasive population haplotypes denoted FL-CO1-01-05 with a Pb- or Pm-prefix. Published haplotypes are Pb-CO1-A ( [Acc. No. KF010492], China), Pb-CO1-B ( Liu et al. (2013) [Genbank Acc. No. KF293729], China), Pb-CO1-C ([Acc. No. JX401103], China), and *P. molurus* Pm-CO1-B (Supikamolseni and Srikulnath 2014 [Acc. No. AB920233], Thailand). The *P. molurus* BOLD published sequence is Pm-CO1-A (BOLD sequence ID: ISDB081-13). Vertical numbers represent polymorphic base pair positions within the alignment and dots indicate sequence homology with the Pb-CO1-A haplotype. Diagnostic sites (N=24) differentiating the two species are underlined.

| \|  \| Polymorphic sites \| \| --- \| --- \| \|  \| 111111112222233344444444555555555555 \| \| Haplotypes \| 29124567891246714900223556012234566688 \| \|  \| 229477387966501682025062362801573214725 \| \| Pb-CO1-A \| TTCTTAAGTTTCTGAAATCGGCCATACTAATTTCGCATG \| \| Pb-CO1-B \| .........C..C.........T......G......... \| \| Pb-CO1-C \| .........C..C................G......... \| \| Pb-FL-CO1-H01 \| ..............G.....A........G......... \| \| Pb-FL-CO1-H02 \| ............C........T.......G......... \| \| Pb-FL-CO1-H03 \| .............................G......... \| \| Pb-FL-CO1-H04 \| ..............C.....A........G......... \| \| Pm-FL-CO1-H05 \| CCTCCGCAC.CT.A.GGCTTA....GTC.G.ACTAT.CA \| \| Pm-CO1-A \| CCTCC.CA..CT.A.GGCTTA..GCG.CGGCACTATGCA \| \| Pm-CO1-B \| CCTCCGCAC.CT.A.GGCTTA....GTC.G.ACTAT.CA \| |
| --- | --- | --- | --- | --- | --- | --- | --- | --- | --- | --- | --- | --- | --- | --- | --- | --- | --- | --- | --- | --- | --- | --- | --- | --- | --- | --- | --- | --- |

**Figure S1.** Bayesian clustering analysis showing *K*=4 for 22 microsatellite loci with 389 python genotypes in Structure 2.4.3. Many of the samples with *P. molurus* haplotypes were assigned to cluster 2 (green). See Fig. 2 in the main manuscript which identified the *K*=2 plot as the ‘correct’ grouping with the largest ∆*K* and smallest *K* overall.

**
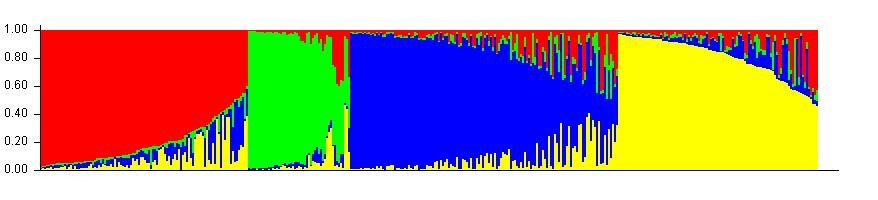
**
